# Intermittent Vertical Diplopia as a Rare Manifestation of a Rare Cerebral Infarct: Artery of Percheron Ischemic Infarct and Sidelights on the Phenotypic Variability of Thalamic Ocular Disorders

**DOI:** 10.7759/cureus.12499

**Published:** 2021-01-05

**Authors:** Hassan Kesserwani

**Affiliations:** 1 Neurology, Flowers Medical Group, Dothan, USA

**Keywords:** stroke, vision disturbance

## Abstract

The thalamus is a complex structure with over 40 named nuclei. Ischemic lesions of the thalamus exhibit a panorama of phenomena ranging from facial numbness to ocular and visual field disturbances to hemiplegia, behavioral disorders, and stupor. It is a dense neuronal hub with a bewildering variety of connections and functions. We present an intriguing case of intermittent vertical diplopia due to an artery of Percheron ischemic infarct of the bilateral paramedian thalami. We seize upon this opportunity to simplify the thalamic nuclei sub-divisions and their vascular supply. In this process, we outline the phenotypic variability of thalamic diplopia and ophthalmoplegia and their various underlying mechanisms.

## Introduction

The thalamus is a very complicated organ with a complex web of interactions and at least 40 named nuclei. Its functions are too many to list. However, traditional anatomists have simplified and stratified thalamus functions. Remarkably, in evolutionary terms, sub-divisions of the cerebral cortex are defined by thalamic afferentation. It is not a trivial fact that the thalamus has no olfactory input, an archaic sensory modality. The thalamus is a later evolutionary development that may have sparked the seeds of consciousness [1]. 

At the outset, in order to streamline its complexity, we will outline the coarse granular functions of the thalamus. The anterior thalamic nuclei project to the limbic cortex and are involved in behavior and personality. Walter Freeman targeted this very tract in his abandoned technique of pre-frontal leukotomy in psychiatric patients in the 1940s. The dorsomedial thalamic nuclei project to the pre-frontal cortex, and damage to them can lead to a Korsakoffian syndrome of confabulation. The ventrolateral nuclei project to the motor and pre-motor cortex and are involved in motor control [2, 3]. Lastly, the pulvinar, lateral and medial geniculate nuclei are involved in visuomotor control, visual and auditory functions, respectively. 

The thalamus derives its name from the Latin term "thalamus", which means "inner chamber or sleeping room". Morphologically, this term is quite apt for a deep-seated and geometrically oval and solid structure. However, metaphorically speaking, the thalamus is a chamber of surprises with a bewildering complexity of functions that at its apogee, along with the temporo-parieto-occipital cortices, may be the spark or engine of consciousness. This explains why diencephalic lesions may lead to stupor and disruptions of thalamocortical oscillations may lead to the impaired states of consciousness associated with absence epilepsy [4], the diencephalon being defined as the thalamus, epithalamus and hypothalamus.

The thalami and midbrain are supplied by branches from the posterior cerebral (PCA) arteries and posterior communicating (PComm) arteries - major arteries of the posterior cerebral circulation. The vascular pattern is relatively simple but highly variable and we will outline them graphically in the discussion section. Certain principles are governing the disease of these blood vessels. The arteries feeding the thalami are perforators and end-arteries with fewer anastomotic connections. They tend to have a higher pulsatility index. Typically the elasticity of the larger arteries absorbs the systolic spike of blood pressure, protecting the smaller downstream arteries. The loss in elastic recoil due to aging, hypertension, and diabetes is due to the arterial wall's stiffness. The smaller diameter of the smaller blood vessels increases their resistance, stiffness, and consequently their pulsatility index. This explains the increased risk of lipohyalinosis, vessel wall thickening and remodeling, and lacunar disease of the thalami [5, 6].

The panorama of thalamic function is also displayed by the phenomenon of diaschisis. The term diaschisis is derived from Greek and means "shocked throughout". Diaschisis refers to a distant collapse in function, perfusion and metabolism of a region distant from the site of injury. Ipsilateral decreased perfusion of the thalamus, as measured by computed tomography (CT) scanning, is frequently found in patients with middle cerebral infarction, highlighting the strong connections of the thalamus with major regions of the brain [7]. 

Normally the medial thalami are supplied on each side by a branch of the PCA ipsilaterally. The artery of Percheron is a branch of the PCA and it is a variant that arises as a solitary trunk supplying both medial thalami and upper midbrain. An ischemic stroke here can cause stupor, agitation, change in behavior, aphasia (dominant side), or hemineglect (non-dominant side). This is similar to the upper diencephalic syndrome as seen with top-of-the-basilar arterial ischemia [8, 9]. Involvement of the upper midbrain can explain ocular findings such as diplopia due to involvement of the interstitial nucleus of Cajal (INC), the largest nucleus of the medial longitudinal fasciculus (MLF). The INC is the neural integrator for vertical eye movement and is involved in vertical gaze, both with saccadic generation and the vestibulo-ocular reflex (VOR) [10].

In a retrospective review of 342 patients with thalamic infarcts, 11.7% of patients demonstrated neuro-ophthalmological findings. In descending order of frequency, these included vertical gaze palsy, skew deviation, third cranial nerve palsy, pseudo-abducens palsy, visual field defects and isolated ptosis or miosis. With a frequency of 84.8%, paramedian thalamic infarct was by far the most common infarct. About 80% of the patients recovered by three months. Those with persistent deficits were more likely to exhibit vertical gaze palsy and concomitant upper midbrain infarcts [11]. 

## Case presentation

We present the case of an 81-year-old healthy and independent woman who developed the sudden onset of painless vertical skew diplopia. The diplopia was intermittent and occurred while driving and when watching television. There was no particular gaze predilection and no worsening with activity or in the evenings. There was no associated numbness, dysarthria, vertigo, or ataxia. The hearing was preserved and free of tinnitus. The whole episode of intermittent diplopia lasted three days. 

Her past medical history was significant for hypertension, treated with extended-release diltiazem at a dose of 360 milligrams (mg) daily. On examination, the blood pressure (BP) was 151/76 with a heart rate (HR) of 78 beats per minute. The height was 5 foot and 3 inches, the weight of 153 pounds, and a body-mass-index (BMI) of 27.5. 

Auscultation of the carotid arteries did not reveal a carotid bruit and cardiac auscultation was free of a murmur.

Her gait stability and cadence were normal, with preserved tandem walking. The pertinent findings on cranial nerve examination are listed. The ocular motion was full in all directions, without nystagmus or fatiguability. Ptosis was not provoked by repetitive blinking. Cogan's eyelid twitch was absent. Visual field testing was full to confrontation. Her speech volume, cadence and quality were entirely normal. Other bulbar functions such as palate elevation was symmetric, pharyngeal sensation intact bilaterally and gag reflex was lively. Genioglossus function was preserved with midline tongue protrusion. The rest of the neurological examination was normal.

Myasthenia gravis serum testing, including acetylcholine receptor binding and blocking antibodies and anti-striational antibodies, were normal. Muscle-specific tyrosine kinase antibodies were also negative. 

**Figure 1:**
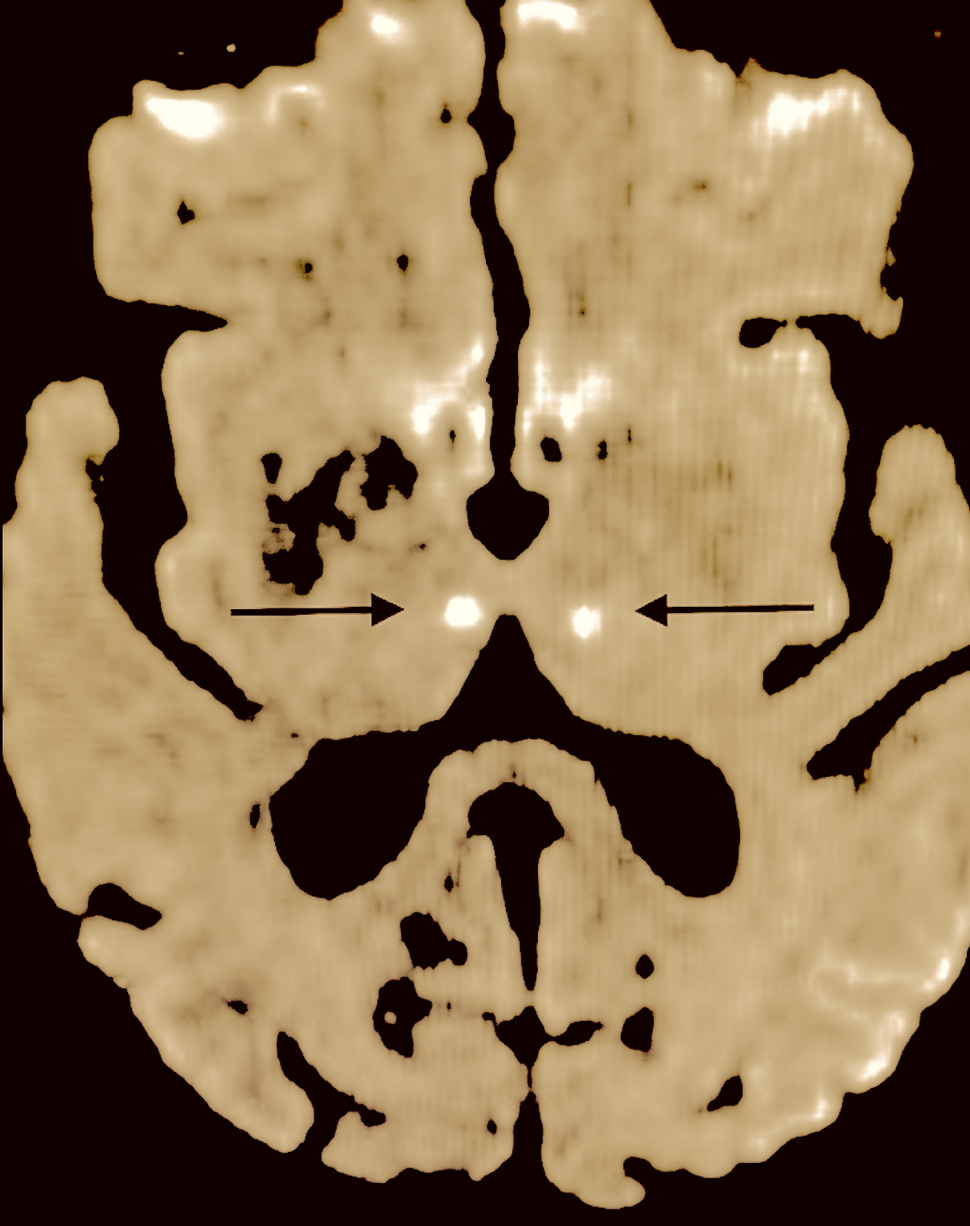
DWI MRI: bilateral mesial thalamic ischemic infarcts implying an artery of Percheron infarct (black arrows) Color, saturation and hue adjusted to highlight infarcts. DWI - diffusion-weighted images

A magnetic resonance imaging (MRI) study revealed bilateral mesial thalamic infarcts on diffusion-weighted imaging (DWI), implicating a solitary trunk of the paramedian arteries perfusing the bilateral mesial thalami, the so-called artery of Percheron (Figure 1).

The patient was started on aspirin 81 mg daily. A carotid duplex scan, 30-day cardiac event monitor, and magnetic resonance angiography (MRA) were normal. Unfortunately, due to the lack of a higher Tesla magnetic field strength, the MRA images were not highly penetrating and were unable to demonstrate the artery of Percheron. Nevertheless, this pattern of bilateral mesial thalamic infarcts is very typical of an artery of Percheron ischemic infarct, and a 3-Tesla strength MRA may have outlined the artery of Percheron. 

## Discussion

**Figure 2:**
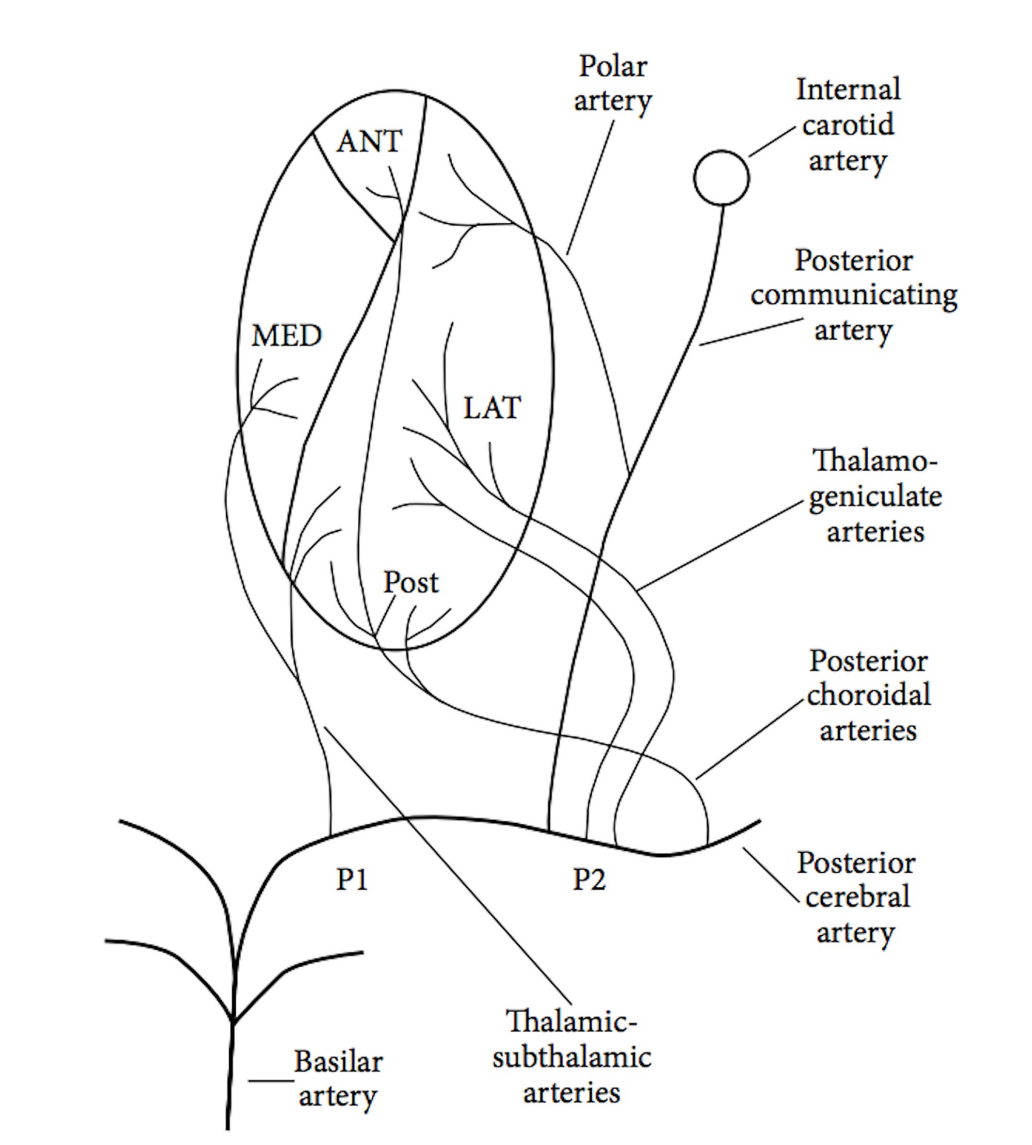
Thalamic vascular territory: branches from the posterior cerebral artery and posterior communicating artery P1, P2 - posterior; ANT - anterior; LAT - lateral; MED - medial; Post - posterior The image is reprinted under the Creative Commons Attribution License. Source: [12]

As outlined earlier, thalamic blood flow is derived from the posterior circulation, namely branches of the PCA and PComm. It should be noted that the posterior choroidal and thalamogeniculate arteries usually arise as a series of branches and not as solitary trunks. The paramedian arteries (formerly known as the thalamic/sub-thalamic arteries) are the only branches arising from the posterior P1-segment of the PCA. The topography of thalamic blood flow is displayed below in Figure 2 [12].

The general outline is that the anterior territory is supplied by the tubero-thalamic arteries, the paramedian territory from the paramedian arteries, the inferolateral territory from the thalamo-geniculate arteries and the posterior territories from the posterior choroidal arteries. 

The clinical manifestations of lesions of the thalamus reflect its widespread connections to the brainstem (disorders of consciousness), upper midbrain (ocular motility disorders), pre-motor cortex (movement disorders), prefrontal lobes (cognition and memory disorders), and parietal lobes (visual disturbances). The arterial branches, the thalamic nuclei perfused and clinical syndromes arising from ischemia to the thalamic nuclei are displayed in tabular form (Table 1) [13].

**Table 1:** Thalamic nuclei, vascular territory, thalamic function and thalamic manifestation of disease PCA - posterior cerebral artery; PComm - posterior communicating artery; P1, P2 - posterior

Blood vessel	Origin	Thalamic nuclei	Function	Clinical presentation	Associated pathways
Tuberothalamic artery	PComm	Reticular, intra-laminar, anterior nuclei, amygdalo-fugal tract, mammilo-thalamic tract	Arousal, autobiographic memory, learning	Drowsiness, stupor, confusion, change in behavior, loss of memory, aphasia (left-sided lesion), hemispatial neglect (right-sided lesion)	Ascending reticular formation and cerebral cortex
Paramedian artery	P1 segment of PCA	Dorso-medial, postero-medial, ventro-lateral	Ocular motor control, arousal, memory	Same as above plus peduncular hallucinosis, akinetic mutism (bilateral), thalamic dementia	Temporal lobes, midbrain ocular centers, frontal eye fields, mammillary bodies
Infero-lateral artery	P2 segment of PCA	Dorso-lateral	Social skills, personality	Apathy, agitation, aggression	Pre-frontal lobes
Principal inferolateral (thalamo-geniculate)	P2 segment of PCA	Ventro-posteromedial, ventro-posterolateral, ventrolateral nucleus, ventro-medial nucleus	Sensation, motor function	Hemi-anesthesia, hemi-ataxia, hemiparesis, post-thalamic pain syndrome (right hemisphere dominance), thalamic arm (main thalamique), choreoathetosis	Parietal lobes
Posterior choroidal artery	P2 segment of PCA	Lateral geniculate nucleus, pulvinar, centromedian nucleus, lateral posterior nucleus	Vision, motor control	Hemianopia, quadrantanopia, dystonia, tremor	Occipital and parietal lobes

**Figure 3:**
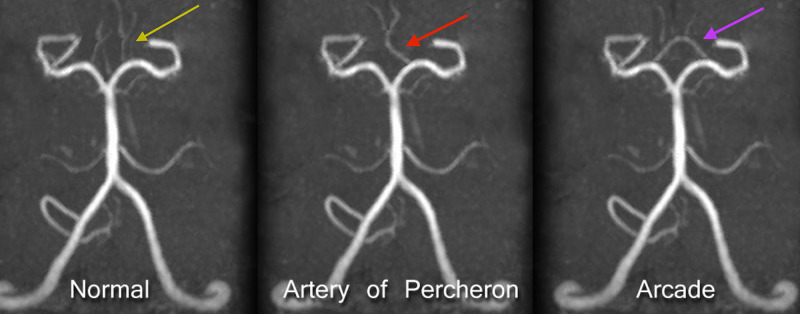
Magnetic resonance angiography: variants of the paramedian artery Ipsilateral origin on each side (yellow arrow), common origin from one isolated trunk supplying bilateral paramedian nuclei (red arrow), arcade pattern (purple arrow). The image is reprinted under the Creative Commons Attribution License. Source: [14]

The paramedian artery from the P1 segment of the PCA has three variants, including the artery of Percheron from an isolated common trunk that supplies the bilateral medial thalami (Figure 3) [14].

The artery of Percheron has four patterns of perfusion, variably supplying the paramedian thalami, anterior thalamus and upper midbrain, thereby determining the clinical phenotype. This is based upon a retrospective study of 37 patients (Table 2) [15]. 

**Table 2:** The artery of Percheron: patterns of perfusion and frequencies

Variant	Frequency
Bilateral paramedian thalami	38%
Bilateral paramedian thalami + anterior thalamus	5%
Bilateral paramedian thalami + upper mid-brain	43%
Bilateral paramedian thalami + anterior thalamus + upper midbrain	14%

In order to understand the complexity of ocular disorders of the thalamus and the upper mid-brain, one can apply certain "rules of thumb". Firstly, the posterior thalamus regulates vergence and goal-directed ocular motion via the pulvinar. Secondly, any insult that increases vertical downward pressure on the thalamus is translated into interruption of sympathetic outflow from the hypothalamus (an autonomic hub), leading to miosis.

We also need to understand the neuroanatomy of the rostral interstitial nucleus of Cajal (rINC) and the pulvinar. The rINC is the largest nucleus of the medial longitudinal fasciculus (MLF). It lies lateral to the Edinger Westphal nucleus (part of the oculomotor nerve complex). The rINC is the neural integrator that generates volitional vertical and torsional eye motion and facilitates the vestibulo-ocular reflex. Meanwhile, the pulvinar is the largest thalamic nucleus, supplied by the posterior choroidal arteries, the P2 segment of the PCA. The pulvinar receives fibers from the superior colliculus and projects to the dorsal visual pathway into the parietal lobes, the "where pathway". Any lesion along this pathway impairs volitional eye motion into the contralateral hemifield, which may appear as a hemineglect or paralysis of contralateral gaze [16]. The pulvinar, paramedian thalami and upper midbrain receive blood supply from the paramedian and posterior choroidal arteries.

In addition to the ocular disorders outlined by the 10-year retrospective study of Moon et al. [11], there are syndromic presentations that can be explained by the proximity of the thalamus to the oculomotor complex, MLF, and rINC. It is also important to note that volitional eye movements begin in the frontal eye fields and that descending pathways synapse in the thalamic nuclei, such as the pulvinar and paramedian thalamic nuclei. These descending pathways are modulatory, inhibitory, or excitatory. The following list is not exhaustive but demonstrates the range and mechanisms of ocular motility disorders (Table 3).

**Table 3:** A tabulation of some of the commoner and less well known ocular motility disorders following thalamic infarcts rINC - rostral interstitial nucleus of Cajal

Study	Ocular disorder	Lesion	Mechanism
Gomez CR, et al. [17]	Pseudoabducens palsy (thalamic esotropia)	Contralateral pulvinar and connecting pathways to oculomotor nucleus	Excessive vergence ocular motion due to loss of inhibitory descending input and overactive medial rectus function
Siatkowski RM, et al. [18]	Vertical gaze palsy	rINC and extension to upper midbrain	Impaired neural integrator for vertical eye motion
Bogousslavsky J, et al. [19]	Vertical " one-and-a-half " gaze palsy	Paramedian thalamus and upper midbrain	Damage to posterior commissure and ipsilateral fibers to inferior rectus and contralateral superior oblique after decussation
Adamec I, et al. [20]	Ocular tilt reaction / Parinaud syndrome	Paramedian thalamus	Involvement of medial longitudinal fasciculus and upper midbrain

## Conclusions

Our case highlights the protean manifestations of lesions of the thalamus. Being a hub and deep-seated nexus, the thalamus interconnects with many and nearly every part of the brain via to-and-fro oscillatory pathways. It is involved in many functions of the brain, including cognition, processing of sensation, motor modulation, vision, balance, and speech. Aberrations of these pathways can lead to diseases as variable as absence epilepsy, post-thalamic pain syndrome, diplopia, or even dementia. Our case also highlights the transient nature of some of these manifestations. Thalamic diplopia, though rare, helps us unmask and understand these pathways better. In our case, simultaneous lesions of the para-median thalami, which is a manifestation of a unique variant of the para-median thalamic arteries, the artery of Percheron, suggests a potential interconnecting pathway of the paramedian thalami to the rINC, the vertical gaze neural integrator. Only further studies such as advanced diffusion tensor tractography may shed further light on this potential pathway.
